# Evaluation of selenium on kidney function following ischemic injury in rats; protective effects and antioxidant activity

**DOI:** 10.15171/jrip.2017.18

**Published:** 2016-11-24

**Authors:** Amin Hasanvand, Abolfazl Abbaszadeh, Saeideh Darabi, Afshin Nazari, Mohammadreza Gholami, Ali Kharazmkia

**Affiliations:** ^1^Department of Pharmacology, Faculty of pharmacy, Lorestan University of Medical Sciences, Khorramabad, Iran; ^2^Department of Surgery, Lorestan University of Medical Sciences, Khorramabad, Iran; ^3^azi Herbal Medicines Research Center, Lorestan University of Medical Sciences, Khorramabad, Iran; Lorestan Veterinary Organization Office, Khorramabad, Iran; ^4^Razi Herbal Medicines Research Center, Lorestan University of Medical Sciences, Khorramabad, Iran; ^5^Department of Anatomical Sciences, Lorestan University of Medical Sciences, Khorramabad, Iran; ^6^Department of Pharmacotherapy, Faculty of pharmacy, Lorestan University of Medical Sciences, Khorramabad, Iran

**Keywords:** Selenium, Antioxidant, Ischemia-reperfusion

## Abstract

**Introduction:** Renal dysfunction is caused by ischemia-reperfusion (I/R) injury, which is a common problem in kidney surgery or kidney transplantation. The human body consists of enormous complex antioxidant systems, which inquires adequate selenium (Se) absorption for normal physiologic function. It is known that Se has some antioxidant effects.

**Objectives:** In the present research, effects of the Se on damages caused by I/R injury investigated.

**Materials and Methods:** In this experimental research, four groups of rats (weighing 220±10 g) used, include control group, I/R group, healthy group treated with Se for two weeks, and I/R group with two-week Se treatment. On the test day, I/R was treated in both right and left renal arteries for 45 minutes and the reperfusion was done for 24 hours.

**Results:** In I/R group, the amount of urea and serum creatinine (Cr) was an injury indicator of the kidney cells which showed a significant increase compared with the control group. When the treatment with Se significantly reduced these indicators, glutathione (GSH) enzyme levels reduced significantly in the second group and the enzyme levels increased due to Se treatment in the fourth group. Furthermore, malondialdehyde (MDA) enzyme levels increased in I/R group due to the Se treatment in the fourth group which was significantly reduced. In addition, the tissue damage was reduced in the fourth group compared with I/R group.

**Conclusion:** Se has a protective effect against the I/R injury. This effect might be due to the antioxidant properties of Se.

**Implication for health policy/practice/research/medical education:**

Ischemia-reperfusion injury is the major etiology of chronic renal failure. Its pathophysiology has been widely studied. Oxidative stress has been known to be involved in it extensively. Thus a couple of antioxidative substances has been studied to figure out if they can modify ischemia-reperfusion injury. In current study we tried to assess a couple of oxidative biomarkers in ischemia-reperfusion injury, following administration of Se to show whether it can amend their changes and reduce Cr and albumin/Cr as well.

## Introduction

Ischemia-reperfusion (I/R) damage in the kidney is frequently met in several clinical states such as hypovolemia following major suprarenal aortal intravenous in renal transplantation (1). In during I/R, reperfusion can cause renal injury in addition to the ischemia (2). In any acute transplantation, any failure in the early stages, delay in transplantation function or very weak function and failure in the early stages of transplantation would have the long-term effects in relation to the ischemic-reperfusion injury (3). The tissue damages due to the I/R injury occur subject to the oxygen free radical release, the mitochondrial dysfunction in the regulation of the intracellular calcium, microscopic cardiovascular dysfunction, failure of blood flow return to the microscopic vessels, and the aggravated inflammatory response associated with immune cell infiltration (4). Reperfusion in the renal is a cause the production of reactive oxygen species (ROS). In the natural conditions, the activity of antioxidant enzymes neutralizes the oxygen free radical concentrations in cells, hence, during reperfusion, the protective ability of these enzymes decreases due to the rapid production of ROS. An excess of ROS cause DNA and cellular damage that leads to apoptosis and cell death. Thus, diminish of the oxidative stresses with drug treatment and/or diet modification would give a good objective for prevention of I/R damage (5). The human body consists of enormous complex antioxidant systems. Selenium (Se) is an essential mineral trace element that has antioxidant properties because of its biological function as a scavenger of free oxygen radicals (6). This element is an essential factor of the antioxidant activity that preserves cells against the effects of free radicals created during normal oxygen metabolism and reduces ROS-mediated signaling pathways (7). The various studies have shown the protective effects of the Se in animal models of cardiovascular diseases, etc. (8,9). The superoxide anion-scavenging effects of six selenocarbamates using a chemiluminescence technique investigated by Takahashi et al (10). In addition, the removal of peroxynitrite (ONOO-) with ebselen and selenomethionine is reported (11-13). Se is an antioxidant may prevent injury during renal I/R by limiting the oxidative injury. This study was designed to examine the anti-oxidative effects of Se after I/R on renal tissue injury.

## Materials and Methods

### Animals

Forty male Sprague-Dawley rats (10-11 weeks) weighing 220 ± 10 g that placed at room temperature of 22±1^o^Cand humidity of 45 ± 10% with 12-hour light/dark cycle were used in this study. The rats were divided randomly into 4 equal groups (n = 10 in each group): 1, control group without I/R (saline); 2, I/R group; 3, control group + Se 0.2 mg/kg; and 4, I/R group + Se 0.2 mg/kg.

In the Se groups, a single dose of sodium selenate (≥98% powder; Aldrich) was injected intraperitoneally (IP) for 2 weeks. All surgical operations were performed under thiopental anesthesia (60 mg/kg), and anesthesia was maintained by additional injections of the same anesthetic.

### Surgical procedure

Briefly, the ultimate region was exposed when the rats were located in the dorsal recumbent position, the abdominal region of rats were shaved and sterilized with povidone iodine solution and a midline laparotomy incision made from superior to the symphysis pubic to the tip of xiphoid process. To observe the kidneys located in the retro peritoneal region, the intestines were removed (14). Then both left and right pedicles were occulted bilaterally with two microvascular clamps (15). The clamps were put bilaterally for 45 minutes so that does not damage to vascular. After the clamps, the color of kidney turned pale and established renal ischemia (16). Forty-five minutes after of ischemia, the clamps removed from zone to consider the kidneys for 5 minutes up to their color turned brown; this change of renal paint confirmed the reperfusion.

### Biochemical evaluations

All tissue specimens were washed with 0.9% NaCl, and a part of kidney tissue was fixed in formalin for histologic examination. That biochemical analyses could be done to determine the tissue levels of malondialdehyde (MDA), glutathione (GSH) and copper-zinc superoxide dismutase (Cu/Zn-SOD). Left kidney was homogenized for 15 minutes at 5000 rpm at 4ºC (17).

### Kidney parameters

The concentrations of blood urea nitrogen (BUN), creatinine (Cr) levels and urine albumin/Cr were measured using according to the standard protocol (18).

### Renal histopathological evaluation

Briefly, for renal histopathological evaluation, the paraffin blocks of the right kidney were cut using a microtome at a thickness of 4 µm, and the sections were stained with Hematoxylin and eosin (H&E). The histopathology sections were examined under a microscope for the presence of tubu­lar necrosis and eosinophilic casts regarded semiquan­titative. The scoring system used for histopathology evaluation of the kidney tissue was as follow: no damage = 0, mild = 1; unicellur patchy isolated, moderate = 2; damage less than 25%, severe = 3; damage between 25%-50%, very severe = 4; more than 50% damage (19).

### Ethical issues

The research followed the tenets of the Declaration of Helsinki. The research was approved by ethical committee of Lorestan University of Medical Sciences. Prior to the experiment, the protocols were confirmed to be in accordance with the Guidelines of Animal Ethics Committee of Lorestan University of Medical Sciences.

### Statistical analysis

Statistical significance was determined by the Kruskal-Wallis test, followed by the Mann-Withney U test as a post hoc test. All data are presented as means ± standard deviation (SD). Results were considered significant for *P* value <0.05.

## Results

### Assessment of the level of MDA

**Figure 1:**
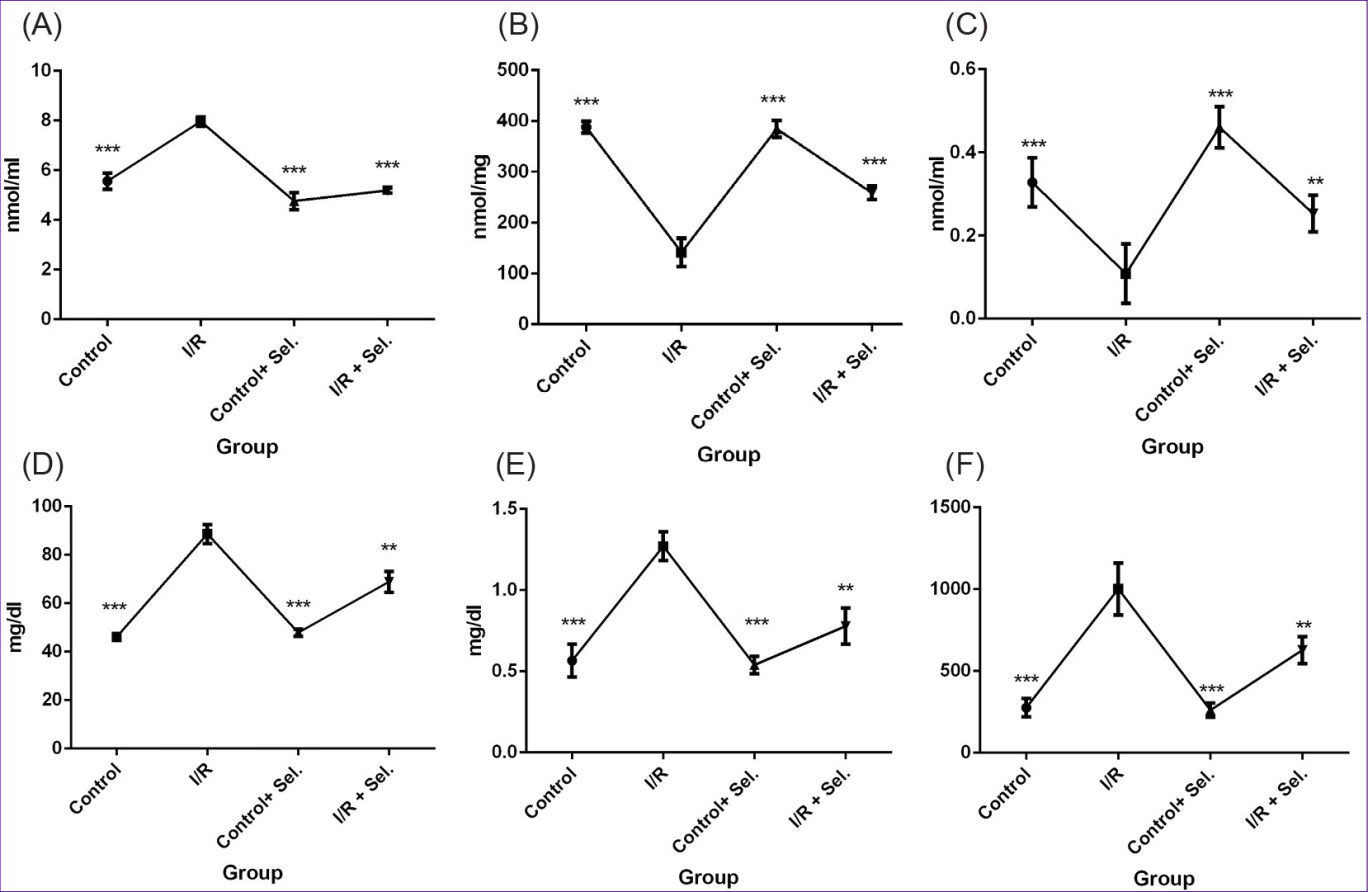
(A) The effects of selenium administration on MDA (n = 10 rats per group). (B) The effects of selenium administration on GSH (n = 10 rats per group). (C) The effects of selenium administration on SOD (n = 10 rats per group). (D) The effects of selenium administration on urea (n = 10 rats per group). (E) The effects of selenium administration on serum Cr (n = 10 rats per group). (F) The effects of selenium administration on Alb/Cr (n = 10 rats per group). Rats were treated with selenium 0.2 mg/kg once daily for a period of 14 days. The data were shown as mean ± standard error of mean (SEM). * P < 0.5, ** P < 0.01 and *** P < 0.001 compared to I/R group.

As shown in Figure 1A the MDA concentrations in I/R group were significantly increased in comparison with saline (control) groups on 2 weeks after I/R induction. Post hoc test analysis demonstrated that administration of Se significantly decreased MDA on days 14 after induction of I/R (Figure 1A).

### Assessment of the level of GSH

GSH was significantly decreased in I/R rats compared to control rats (saline) on two weeks after I/R induction. Moreover, our results showed that administration of Se increased GSH in a significant manner in days 14 after induction of I/R in comparison with I/R group (Figure 1B).

### Assessment of the level of SOD

Figure 1C shows the results of SOD assessment. As shown, the level of SOD significantly decreased in the serum of I/R animals in compared to control (saline) groups on days 14 after induction of I/R in rats. Results showed that Se increased the concentration of SOD in I/R rats on days 2 weeks after induction of I/R when compared to I/R group (Figure 1C).

### Assessment of the level of serum urea

Analysis showed that the level of urea significantly decreased in I/R rats in comparison with control ones on days 14 after induction of I/R. Furthermore, our findings showed that administration of Se in day 14 after I/R induction decreased the urea levels in comparison with I/R group (Figure 1D).

### Assessment of the level of serum creatinine

Analysis of variance (ANOVA) analysis showed that the level of Cr significantly increased in I/R rats in comparison with control (saline) animals on days 14 after I/R induction. Post hoc test analysis showed that, Se in days 14 after I/R induction significantly reduced the levels of Cr in I/R group (fourth group) when compared to I/R groups (Figure 1E).

### Assessment of the level of albumin/Cr

Figure 1F shows the results of albumin/Cr assessment. As shown, the level of albumin/Cr significantly increased in the I/R animals in compared to control (saline) groups on days 14 after induction of I/R in rats. Results showed that administration of Se reduced the concentration of albumin/Cr in the I/R rats on 2 weeks after induction of I/R (Figure 1F).

### Selenium decreases tubular necrosis and eosinophilic casts

Tubular necrosis significantly increased in I/R group compared with the control group. The treatment with Se could reduce tubular necrosis compared with I/R group in the fourth group. Tubular distension in the I/R group is significantly decreased in the fourth group that was treated with Se. Eosinophilic casts in the second group (I/R) was significantly increased compared to the control group, while the casts were decreased in the fourth group with Se treatment (Table 1).

**Table 1:** The mean values of total tissue damage scores in groups

**Experimental groups**	**Parameters**		
	**Tubular necrosis**	**Eosinophilia casts**	**Tubular dilatation**
Control	0.41 ± 0078	0.38 ± 0.054	62.89 ± 0.03
I/R	1.24 ± 0.032^a^	1.41 ± 0.122^a^	89.73 ± 3.92^a^
Control+ Se	0.75 ± 0.08^ab^	0.64 ± 0.089^ab^	71.347 ± 3.81^b^
I/R + Se	0.8 ± 0.082^b^	0.49 ± 0.058^b^	67.17 ± 2.79^b^

Data are presented as Mean ± SEM, *P* < 0.05 was considered to be statistically significant. ^a^ *P* < 0.05 as compared with control. ^b^ *P* < 0.01 as compared with I/R.

## Discussion

Results showed that administration of Se notably decreased MDA, serum urea and Cr in comparison to untreated I/R rats. Also, results showed that treatment with Se increased GSH and SOD levels in I/R rats.

In this study, the biochemical and histological examination showed that administration of Se decreased renal injury following I/R and lipid peroxidation. The results from the recent studies suggest that Se can help to protect tissues against oxidative damage. In this study, it was found that Se has antioxidant protective effects on the kidney tissue and it prevents apoptosis. These results were in agreement with our histological observations. I/R may induce systemic and/or local damage in the functional valence (20). In addition, ischemia is well-known to elevate the ROS after perfusion, and serving as an important effector in tissue injury (21). The results of this study have indicated that ischemia during 45 minutes and the post 24-hour reperfusion renal tissue cause injury in kidney and lead to renal dysfunction in rats. Antioxidant free radical scavengers are the mediators responsible for ischemic damage may be used to prevent tissue damages (22). The present research has shown, that Se could have a protective effect against I/R injury in the renal. This was observed an increase in the activity of the antioxidant enzymes and less histopathological damage in the rats that had received Se at least 2 weeks and I/R occurred in them. The Se treatment group of rats with I/R (fourth group) showed reduced levels of the serum Cr and urea compared with the second group. Cr and urea are indicators of the kidney structure and when the kidney structure becomes damaged, the levels of these enzymes increased (23). These results agree with several studies have shown that antioxidants may reduce BUN serum levels and Cr (24,25). Several studies have shown that ROS can damage the epithelial cells and induce apoptosis. Two antioxidant systems exist in the body, first; preventive the antioxidant system, including many proteins, e.g. ceruloplasmin, albumin, which are related to the metals and prevent ROS and thus any further chain reaction. Second; the cleaning antioxidant enzyme system, e.g. vitamin E, vitamin C and GSH peroxidase that remove produced ROS, so plasma membrane lipid peroxidation is prevented (26). GPx (Selenium-dependent glutathione peroxidase) is one of the main antioxidative enzyme in the cells (27) and it has been shown that Se is a structural component of GSH peroxidase (28). Se is found in the form of selenocysteine in the active moiety as a consisting component of 25 types of selenoprotein, such as GPx p-selenoprotein (29,30). Se is able of diminishing the production of the oxygen free radicals (31). Se supplement with Na_2_Se_3_ can protect the heart of the immature rats from ischemia and reperfusion injury (9). The function of GPx enzymes as antioxidant reduces peroxides, e.g. H_2_O_2_. It has been shown that the catalytic cycle of glutathione peroxidase 4 (GPx) contains selenic acid, which reacts with GSH to generate selenenyl-sulfide adduct and it decreases peroxide (32). SE is diminished significantly in the blood, serum, and red blood cell (RBC) compared with the control group, following the progression of chronic kidney disease (33). In this study, the antioxidant enzymes levels, e.g. GSH, in the I/R group has decreased significantly as compared with the control group, which is consistent with the other studies (17). The results showed that GSH enzyme level increased significantly in the I/R group receiving Se, compared with the second group. MDA, formed during lipid peroxidation, is a secondary product of oxidative damage and, a marker of tissue injury. Avlan et al found that Se significantly decreased MDA levels in a rat model of testicular torsion/detorsion (34). Administration of Se with strong antioxidant properties led to a fall in MDA levels in tissue samples and a decrease in lipid peroxidation (34). It has been shown that improved SOD level in renal diminished I/R-induced tubular injury and enhanced kidney function after ischemic (35). Inducing SOD production was accompanied by decrease free radical and it was causing attenuates renal failure in post-ischemic (36). A recent study showed that Se may increase SOD production through induction of auto-oxidation in rats (37). Also in lead intoxication and gentamicin-induced acute renal failure, Se has been shown to have renoprotective effects. In fact, it has been shown that Se is reduced associated with the renal disease development process (38). Se has been shown to inhibit injury induced by free radicals to the fatty acid of the subcellular membrane (39).

In conclusion, administration of Se reduced oxidative damage, both histopathological and biochemically, in the early stages of the kidney I/R in our rat model. These data provide support for a pathogenic role of ROS in ischemic renal injury.

## Acknowledgments

We would like to thank all colleagues expert in medicinal plant research center, and all those who have helped us in this study.

## Authors’ contribution

AH, AA,SD and AN conducted the research. AK designed and supervised the study, analyzed the data and prepared the final draft of the article. MG supervised and analyzed the pathology data.

## Conflicts of interest

The authors declared no competing interests.

## Ethical considerations

Ethical issues (including plagiarism, data fabrication, double publication) have been completely observed by the authors.

## Funding/Support

This study was supported by Lorestan University of Medical Sciences with (Grant #A-10-1758-2).
